# Multi-modality imaging in cardiac ATTR amyloidosis: agreement between echocardiography, MRI and DPD-scintigraphy

**DOI:** 10.1186/1750-1172-10-S1-O17

**Published:** 2015-11-02

**Authors:** Ludivine Eliahou, Renata Chequer, Phalla Ou, Vincent Algalarrondo, Teresa Antonini, Michel Slama, Dominique Le Guludec, François Rouzet

**Affiliations:** 1 Hopital Antoine Béclère, Cardiologie, French Referent Center for Rare Diseases for FAP (Familial Amyloid Polyneuropathy) (NNERF), 92140, Clamart, France; 2 Hopital Bichat-Claude Bernard, Médecine Nucléaire, 75018, Paris, France; 3 Hopital Bichat-Claude Bernard, Radiologie, 75018, Paris, France; 4 Hopital Antoine Béclère, Cardiologie, French Referent Center for Rare Diseases for FAP (Familial Amyloid Polyneuropathy), 92140, Clamart, France; 5 Hopital Paul Brousse, Centre Hépato-Biliaire, 94800, Villejuif, France; 6 Hopital Antoine Béclère, Cardiologie,French Referent Center for Rare Diseases for FAP (Familial Amyloid Polyneuropathy), 92140, Clamart, France; 7 Hopital Bichat-Claude Bernard, Médecine Nucléaire, 75018, Paris, France; 8 Hopital Bichat-Claude Bernard, Médecine Nucléaire, 75018, Paris, France

## Background

Three main imaging techniques are commonly used to identify cardiac transthyretin (ATTR) amyloidosis: echocardiography, MRI and DPD scintigraphy. Each one provides specific diagnostic and prognostic informations but also has its specific limitations. We sought to evaluate a multimodality imaging strategy to diagnose cardiac amyloidosis in ATTR.

## Methods

Seventy seven consecutive patients with multimodality imaging evaluation (echocardiography, 1.5T MRI and 99mTc-DPD scintigraphy) to diagnose cardiac amyloidosis were identified from the database of the French National Reference Center for Amyloidosis. Patients with pacemaker or severe renal failure did not undergo cardiac MRI and were analyzed on the basis of the echocardiography and scintigraphy (n=17). Three groups were compared: patients with positive agreement to diagnose cardiac ATTR (PA-ATTR group), patients with positive agreement to exclude cardiac ATTR (PA-normal) and patients with negative agreement (NA).

## Results

The mean age was 52 years [44-70]; 59% were male. Transthyretin mutations were Val30Met in 67%, other in 21%, and 12% had acquired ATTR from previous domino liver transplantation; 30 patients had a positive echocardiography, 37 positive MRI and 36 positive DPD scintigraphy. Positive imaging agreement was encountered in 50/77 patients (65%: 30 PA-ATTR and 20 PA-normal). Negative agreement was observed in 27/77 patients (35%). Compared with PA-ATTR patients, NA patients were younger (68 [64-72] years vs. 46 [41-64], had lower BNP levels (149 [94-248] pg/ml vs. 40 [25-102],) and thinner interventricular septum (17 [14-20] mm vs. 12 [10-14]), all p values <0.0001). The two main causes for negative agreement between techniques was the sole positivity of the MRI (n=10) and the sole negativity of the DPD scintigraphy(n=6). Compared with PA-ATTR patients, patients with a sole MRI positivity were younger (41 [39-44] years vs. 68 [64-72] p<0.001), more frequently women (80% vs.26% p=0,002), had thinner interventricular septum (9 [8-11] mm vs.17 [14-20], p<0.0001), had lower BNP levels (26 [24-36] vs 149 [92-249] p<0.0001) and had less diffuse late gadolinium enhancement pattern (10% vs. 66% patients; p<0.0001). As compared with PA-ATTR patients, patients with a sole negativity of the DPD scintigraphy had acquired ATTR from domino liver transplantation in all but one case (83% vs. 6% patients; p=0.02).

## Conclusions

In transthyretin amyloidosis, the agreement between echocardiography, cardiac MRI and DPD scintigraphy to diagnose cardiac amyloidosis was observed in 65% of patients. Patients without agreement between these three techniques had distinct patterns of cardiac involvement: sole positivity of the MRI was encountered in patients in the early stages of ATTR; patients with acquired ATTR due to domino liver transplantation often had negative DPD scintigraphy.

